# Chronic fungal osteomyelitis of the tibia due to *Acremonium curvulum*: a rare case

**DOI:** 10.11604/pamj.2019.34.173.13737

**Published:** 2019-12-03

**Authors:** Sadia Khan, Anil Kumar, Vadakekoottu Bhaskaran, Sabthami Chandran, Kavitha Dinesh

**Affiliations:** 1 Department of Microbiology, Amrita Institute of Medical Sciences, Amrita University, Kochi, Kerala, India; 2 Department of Orthopaedics, Amrita Institute of Medical Sciences, Amrita University, Kochi, Kerala, India

**Keywords:** *Acremonium curvulum*, fungal osteomyelitis, voriconazole for fungal osteomyelitis, tibial osteomyelitis, *Acremonium* spp

## Abstract

Fungal osteomyelitis is a rare disease which usually presents in an indolent manner. Opportunistic infections due to other non-aspergillus moulds are an emerging entity. We report a case of fungal osteomyelitis due to *Acremonium* spp in an immunocompetent adult which showed a chronic, indolent course but responded well to treatment with voriconazole. This case highlights the importance of diagnosing the causative agent in fungal osteomyelitis as species specific susceptibility can aid in the treatment of fungal osteomyelitis.

## Introduction

Fungal osteomyelitis is a rare disease which usually presents in an indolent manner. It affects both immunocompetent and immunocompromised individuals. The possible ways to acquire fungal osteomyelitis include direct inoculation post traumatic injuries contaminated with soil, open wounds or surgery, hematogenous spread or extension from a contiguous site of infection [1]. A majority of these infections are caused by *Candida* spp and *Aspergillus*. Dimorphic fungi and *Cryptococcus* spp are also implicated as etiological agents of fungal osteomyelitis. Opportunistic infections due to other non-aspergillus moulds are an emerging entity [2, 3]. As they are reported infrequently, their mechanism of infections and clinical outcomes remain poorly described. We report a case of fungal osteomyelitis due to *Acremonium* spp in an immunocompetent adult which showed a chronic, indolent course but responded well to treatment. Fungal osteomyelitis due to *Acremonium* spp are rarely reported with three cases previously reported in transplant recipients [4-6].

## Patient and observation

A 59 year old male was admitted to the orthopaedic ward with complaints of multiple draining sinuses in the right calf since 3 weeks. He gave history of 2 episodes of similar draining sinuses at a site above the current sinus which occurred 15 years back. At that time he had not sought any treatment and the sinus had healed by itself. Currently, the patient complained of severe pain and bloody discharge from the sinuses. He gave history of taking antibiotics prescribed by local physicians. There was no history of trauma, fever, chronic cough or loss of weight and appetite. The patient was a diabetic since the past ten years and was on oral hypoglycaemic agents. On examination of the right leg, two sinuses were present on the anterior aspect of the tibia in the proximal 1/3^rd^ portion. Blood stained purulent discharge was observed in both these sinuses. Another sinus was observed in the posterior calf region, without any discharge. Tenderness was elicited around the sinuses. No neurovascular deficits were observed and distal pulse and movements were preserved. X-ray of the right lower limb showed cortical thickening of the right tibia in the proximal aspect. Magnetic resonance imaging (MRI) with contrast showed enhancing soft tissue mass in medial aspect of tibia in right upper leg in subcutaneous plane which was suggestive of soft tissue infection. In view of the chronic draining sinus, a wound debridement and curettage was done. The intra-operative tissue was sent for histopathology and culture. The patient was discharged on tablet cefuroxime 500mg, with advice to avoid weight bearing on the affected limb.

Histopathology of the tissue showed hyalinised fibro-collagenous tissue with multiple granuloma formation and necrosis. Sheets of macrophage collection and lympho-plasmacytic infiltration were also seen. Many areas showed collections of neutrophils around circumscribed pink eosinophilic material with few filamentous structures suggestive of fungal organism which were PAS & GMS positive. The histopathological picture was suggestive of fungal osteomyelitis with granulomatous reaction. Fungal culture on Sabouraud’s Dextrose Agar grew slow growing colonies of a filamentous fungi which were moist at first then became powdery grey in colour. Hyphae were hyaline with erect phialides and conidia were aggregated at the slimy head of these phialides. The organism was identified as *Acremonium* spp and sent for further identification to a reference centre (VP Chest Institute, New Delhi). The organism was identified as *Acremonium curvulum* by the reference laboratory. The patient was started on oral voriconazole 200mg bd before the fungal identification reports were available. As there was good response to voriconazole initially and the patient was not willing for intravenous therapy; it was continued for 6 weeks initially and prolonged to 6 months. The patient recovered completely and no new sinuses were observed till his last visit which was 24 months after treatment was initiated.

## Discussion

Fungal osteomyelitis was considered a rare entity till recently [7]. The salient risk factors for fungal osteomyelitis include drug induced immunosuppression (protracted chemotherapy-induced neutropenia), corticosteroid therapy, surgical intervention, use of illicit intravenous drugs, broadspectrum antibiotics use, indwelling catheters, diabetes mellitus, HIV infection, organ transplantation and total parenteral nutrition [8]. The patient described in the current case was a diabetic on oral hypoglycaemic agents for 10 years. While he did not remember any trauma leading to sinus development, it appeared to be the most likely source of acquiring the pathogen. Unlike *Candida* and *Aspergillus osteoarticular* infections, which occur as a result of hematogenous spread, direct inoculation is the cause of infection for the majority of non-*Aspergillus filamentous* fungal species reported in literature [1, 9]. Fungi rarely cause disease in healthy immunocompetent hosts; most of the disease results when fungi accidentally penetrate host barriers or when immunologic defects exist, which promote fungal entry and infection. The etiologic agents gain entrance through transcutaneous puncture wounds, usually by a thorn or a splinter, or other kinds of trauma, such as road accident fractures, can be identified as the portal of entry of the fungus [10]. The patient had been seen by multiple local physicians who had prescribed numerous courses of antibiotics. While the antibiotics provided temporary relief, the fungal pathogen spread unchecked causing fungal osteomyelitis of the tibia.

Acremonium spp are unusual causes of fungal osteomyelitis. Few cases of Acremonium osteoarticular infections have been identified in immunocompromised patients with infections of vertebra, bone and knee [11]. Secondly the organism is morphologically and clinically indistinguishable from Fusarium spp during its early phase of growth [12]. Invasive Acremonium infections are secondary to immunosuppression due to malignancy, medication and transplantation [13]. Since Acremonium spp are common environmental saprophytes, it is essential to confirm the infection by histopathology. As seen in our case, the histopathological picture was suggestive of chronic fungal osteomyelitis. Treatment of non-aspergillus osteoarticular infections usually involves combined surgical and medical approach [14] .Optimal antifungal therapy for Acremonium infections is still debatable due to the paucity of reported cases. Amphotericin B, ketoconazole, itraconazole, fluconazole, 5-fluorocytosine, voriconazole and combinations of these antifungal drugs have been tried with variable success [6]. Amphotericin B has been used to treat serious infections in most cases; patients with poor response to amphotericin B have shown resolution with voriconazole [15, 16]. In the described case, voriconazole showed good response initially and was continued for 6 months. The patient remained asymptomatic during his last visit to the hospital, which was 24 months after his therapy was completed.

## Conclusion

This case highlights the importance of diagnosing the causative agent in fungal osteomyelitis. Species specific susceptibility can further aid treatment in fungal osteomyelitis. Voriconazole treatment as seen in this case shows good response and is easier to administer during the prolonged course of treatment required for fungal osteomyelitis.

## Competing interests

The authors declare no competing interests.
